# Further Stabilization of Alcalase Immobilized on Glyoxyl Supports: Amination Plus Modification with Glutaraldehyde

**DOI:** 10.3390/molecules23123188

**Published:** 2018-12-03

**Authors:** Fouzia Hussain, Sara Arana-Peña, Roberto Morellon-Sterling, Oveimar Barbosa, Sabrina Ait Braham, Shagufta Kamal, Roberto Fernandez-Lafuente

**Affiliations:** 1 Departamento de Biocatálisis, ICP-CSIC, Campus UAM-CSIC, Cantoblanco, 28049 Madrid, Spain; fouziagcu@gmail.com (F.H.); sara_arana@hotmail.com (S.A.-P.); roberms@gmail.com (R.M.-S.); sabrina.aitbraham@yahoo.fr (S.A.B.); 2 Department of Biochemistry, Government College University, Faisalabad 38000, Pakistan; shaguftakamal@gcuf.edu.pk; 3 Departamento de Química, Facultad de Ciencias. Universidad del Tolima, Ibagué 546, Colombia; oveimar@gmail.com; 4 Laboratoire de Biotechnologies Végétales et Ethnobotanique, Faculté des Sciences de la Nature et de la Vie, Université de Bejaia, Bejaia 06000, Algeria

**Keywords:** enzyme immobilization, enzyme stabilization, solid phase chemical modification, enzyme amination, glutaraldehyde, crosslinking

## Abstract

Alcalase was immobilized on glyoxyl 4% CL agarose beads. This permitted to have Alcalase preparations with 50% activity retention versus Boc-l-alanine 4-nitrophenyl ester. However, the recovered activity versus casein was under 20% at 50 °C, as it may be expected from the most likely area of the protein involved in the immobilization. The situation was different at 60 °C, where the activities of immobilized and free enzyme became similar. The chemical amination of the immobilized enzyme or the treatment of the enzyme with glutaraldehyde did not produce any significant stabilization (a factor of 2) with high costs in terms of activity. However, the modification with glutaraldehyde of the previously aminated enzyme permitted to give a jump in Alcalase stability (e.g., with most than 80% of enzyme activity retention for the modified enzyme and less than 30% for the just immobilized enzyme in stress inactivation at pH 7 or 9). This preparation could be used in the hydrolysis of casein at pH 9 even at 67 °C, retaining around 50% of the activity after 5 hydrolytic cycles when the just immobilized preparation was almost inactive after 3 cycles. The modified enzyme can be reused in hydrolysis of casein at 45 °C and pH 9 for 6 cycles (6 h) without any decrease in enzyme activity.

## 1. Introduction

Proteases are enzymes with many uses in biotechnology, from hydrolysis of proteins to reduction of allergenicity or production of bioactive peptides, to fine chemistry (production of dipeptides, resolution of racemic mixtures, etc.) being the detergent industry the main consumer of proteases [1,2,3,4,5,6,7,8]. Alcalase, a protease solution supplied by Novozyme, has subtilisin Carlsberg as main component and it is produced by a strain of *Bacillus licheniformis*. Although Alcalase has been specifically optimized for use in the alkaline pH range as component of bioactive detergents, this enzyme has been used for different purposes [9,10,11,12,13,14,15,16,17,18,19].

Immobilization of proteases, as of most enzymes, is a requirement for many of the industrial biocatalytic applications, as the process profitability implies the reuse of this relatively expensive biocatalyst [20]. A proper immobilization may greatly improve enzyme performance. One of the most pursued objectives of immobilization is the increase of enzyme stability, via multipoint or multisubunit covalent attachment, although also enzyme activity (mainly under harsh conditions), specificity or selectivity, resistance to inhibitors and chemicals or enzyme purity may be improved via a proper immobilization [21,22,23,24,25,26,27,28,29]. Moreover, it has been recently described how diffusion limitations generated via immobilization on porous supports may be useful in tailoring protease selectivity [30,31]. Using proteases that are intended to be employed in the hydrolysis of proteins, the large size of the substrates needs to be also considered [20]. This makes compulsory to have a proper orientation of the active centre of the enzyme, which should be oriented towards the medium, otherwise the enzyme may become inactive just by steric reasons even having the structure of the active centre unaltered [32]. However, as proteases may have many uses, these biocatalysts will be useful for reactions involving small substrates, for example, in fine chemistry [20]. Alcalase has been immobilized in different supports [33,34,35,36] and among them, Alcalase immobilized on glyoxyl agarose beads has very good properties and has been used in diverse reactions [37,38].

Another strategy of improving enzyme stability is via chemical modification [39,40,41,42]. This modification can change the overall physical features of the enzyme surface, or it can permit enzyme rigidification via intramolecular or intersubunits crosslinkings. The effect of these modifications on previously immobilized-stabilized enzymes couples to the advantages of simplicity of solid-chemistry, the previous stabilization of the immobilized enzyme [43,44]. This paper shows the effect of some chemical modifications of glyoxyl-Alcalase.

A first example has been the use of glutaraldehyde. This bifunctional reagent is very reactive and the modification of the enzyme will yield one point modification of Lys and terminal amino groups (producing unpredictable results in enzyme properties), together with some intramolecular crosslinkings if there are primary amino groups at the proper distance, producing the rigidification of the enzyme structure [45,46,47,48]. It has been shown that optimal reactivity may be achieved using amino-glutaraldehyde reacting versus another amino-glutaraldehyde [49]. The objective will be to get an intense intramolecular crosslinking but this will depend on the amount and distance between reactive groups in the protein.

The second modification assayed has been the full modification of the external carboxylic groups using ethylenediamine (EDA) plus carbodiimide (EDC), a strategy utilized in many instances to modify enzyme surfaces for different purposes [50]: improving the immobilization of enzymes via multipoint covalent attachment [51,52,53,54,55], improving enzyme properties of free or immobilized enzymes [56,57,58,59,60,61], easing preparation of crosslinked enzyme aggregates [62], facilitating a second modification [63], or the promotion of a more intense intramolecular crosslinking using some bifunctional amino reactive reagent [49]. If the amination of the enzyme is not very negative for the enzyme activity/stability, it may be a way of increasing the prospects of achieving an intense intermolecular crosslinking, as the number of reactive groups will be greatly increased after amination. Thus, a further studied modification has been the glutaraldehyde treatment of previously aminated enzymes. This strategy in some instances has permitted to improve enzyme stability [49], while in other cases it has driven to the full enzyme inactivation [57].

The modified immobilized Alcalase activities versus a large (casein) and small synthetic substrate (Boc-l-alanine 4-nitrophenyl ester, NPA) have been studied, as well as the effect of the pH and Temperature in the hydrolysis of casein under different conditions.

This way, the objective of this paper is to analyse the possibilities of getting an intramolecular crosslinking using glutaraldehyde in immobilized Alcalase and how these possibilities may be modulated if the enzyme is previously aminated.

## 2. Results

### 2.1. Immobilization of Alcalase in Glyoxyl Agarose (Glx-AL)

**Figure 1:**
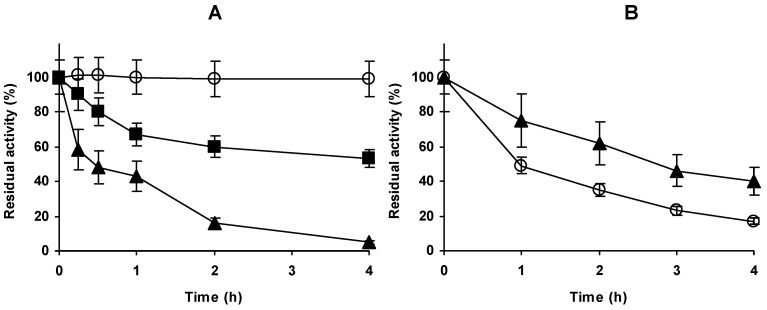
(**A**) Immobilization course of Alcalase in 4% glyoxyl agarose beads. Immobilization was performed at pH 10 and 25 °C as described in methods. Open circles: reference; Solid triangles: supernatant; Solid squares; suspension. (**B**) Inactivation courses of free (open circles) and immobilized (closed triangles) Alcalase. Inactivation was performed at 54 °C and pH 7. Other details are stated in methods section.

**Figure 2:**
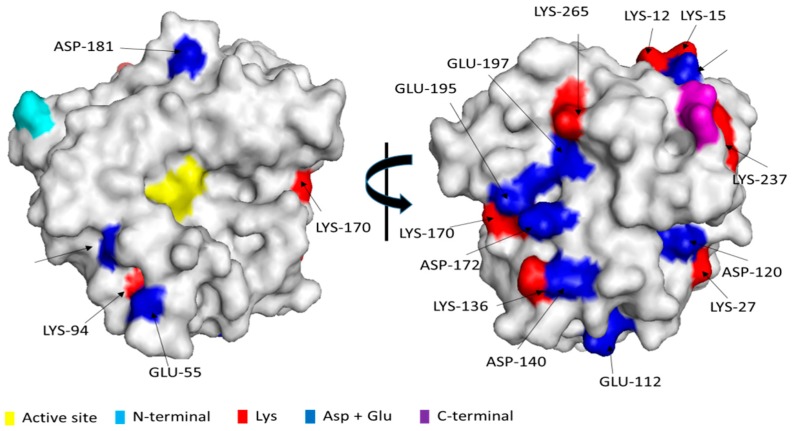
3D surface structure models of Alcalase (PDB code 1SBC). The 3D structures were obtained from the Protein Data bank (PDB) and displayed using Pymol software version 0.99rc6.

Figure 1 shows the immobilization course of Alcalase on 4 BCL glyoxyl-agarose beads. After 4 h most enzyme activity was immobilized and expressed activity was over 50%. This immobilization rate was lower than that found using other enzymes [64]. Considering that the first enzyme immobilization in this support is via a multipoint immobilization, this suggested that the enzyme-support reaction is not very intense, perhaps by a low content in Lys groups in the enzyme surface or by a homogenous distribution [64,65]. As expected from the immobilization rate, the enzyme was only moderately stabilized by the immobilization when compared to the free enzyme (Figure 1B), increasing the half-life by a factor lower than 3. This stabilization promoted by the immobilization may be at least partially caused by the prevention of the enzyme autolysis when in soluble form. Figure 2 shows that the enzyme is not very rich in Lys residues. Thus a high stabilization via multipoint covalent attachment in glyoxyl agarose 4BCL may be quite difficult. Considering that the first immobilization on glyoxyl supports is required to be multipunctual [64], and following Figure 2, immobilization of Alcalase on glyoxyl agarose very likely involves the upper opposite side of Alcalase, where Lys 12 and 15 are very exposed and Lys 265 and 237 can also be part of the immobilization versus a flat surface, as they are relatively near. If the immobilization is via these residues, the active centre of the enzyme will not be fully blocked by the support wall but neither will it be fully accessible for large molecules [66,67]. This means that the orientation of the enzyme to hydrolyse large proteins may not be optimal but it may be good enough to be used in the hydrolysis of casein, as it can be seen in the next point and in literature [37,38]

### 2.2. Effect of Temperature on the Hydrolysis of Casein by Different Alcalase Preparations

**Figure 3:**
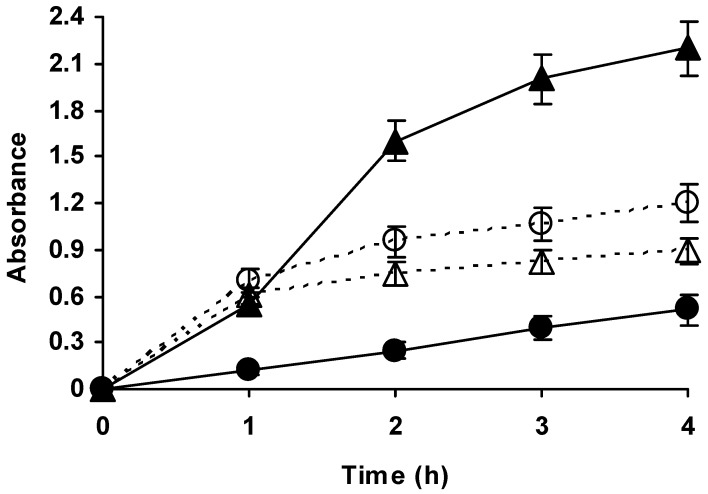
Courses of hydrolysis of casein at pH 9 and 50 (circles) or 60 °C (triangles) catalysed by free (empty symbols, dashed line) or immobilized Alcalase (solid symbols, solid line). The concentration of enzyme (free or immobilized) was 0.005 mg/mL in all cases. Other conditions may be found in methods section.

Figure 3 shows the hydrolysis of casein catalysed by immobilized and free Alcalase at different temperatures. At pH 9 (optimal pH for this reaction using both free and immobilized Alcalase, unpublished data) and 50 °C, the specific activity of the free enzyme was almost five folds higher than that of the immobilized enzyme. Considering the very low loading (around 2.5% of the maximum loading) and low activity of the immobilized enzyme, diffusional limitation may be ruled out. This low protease activity of the immobilized enzyme will fit with the steric hindrances promoted by the support surface to the entry of the casein substrate to the active centre of the enzyme, if the immobilization was performed via Lys 12 and 15, the likeliest orientation considering the multipoint immobilization mechanism of glyoxyl supports [64]. However, at 60 °C the initial activities become similar for both free and immobilized enzyme; while the free enzyme is slightly less active under these conditions, the immobilized enzyme greatly increase the activity (by a factor of 4). The activity of the free enzyme decrease very slightly, in fact in the first point the difference between 50 and 60 °C is not significant and become clearer when the reaction progress (very likely, because this give more time to the free enzyme to become inactivated). Temperature plays a complex role in the hydrolysis of proteins. On the one hand, a higher temperature should increase the catalytic efficiency of the protease. On the other hand, temperature may induce distortion of the protease driving to the enzyme inactivation and facilitating autolysis. Moreover, temperature may also distort the substrate casein, making its proteolysis and the release of fragments of the substrate protein easier that, due to their small size, may be more accessible to the immobilized enzyme molecules. Thus, the results at 60 °C may be due to the fact that the deleterious effect of this temperature on the free enzyme structure is higher than the gain in catalytic efficiency, while this negative effect is lower on the immobilized enzyme (distortion will be decreased and autolysis will be impossible) and the gain in catalytic efficiency is observed [23]. Moreover, the higher temperature facilitates a certain flexibility of the casein molecule, more important for the not perfectly oriented Alcalase molecules than for the free enzyme. In fact, between the first and second hour there is a significant increment on peptides accumulation rate when using the immobilized enzyme (by a factor of around 2). This may be due to the availability of smaller protein fragments, which can be more accessible to the not fully properly oriented protease molecules. After 4 h of reactions, the level of casein hydrolysis was much higher using the immobilized enzyme than using the free enzyme even at 50 °C.

Thus, this immobilized biocatalyst, as previously described [37,38], could be used in the hydrolysis of proteins, however using agarose 4 BCL the stabilization was relatively low, although this permitted an improved performance of the enzyme at 60 °C when compared to the free enzyme.

### 2.3. Modification with Ethylenediamine (EDA) of Alcalase Immobilized in Glyoxyl Agarose Beads (Glx-AL-EDA)

**Figure 4:**
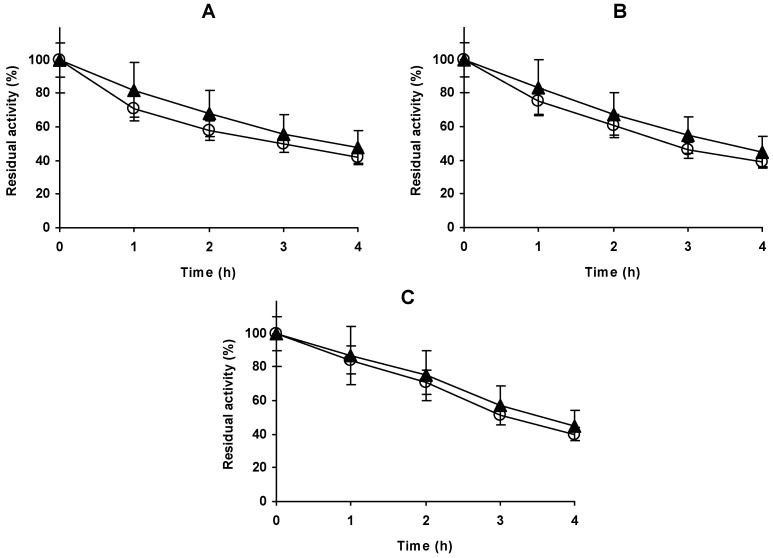
Effect of the amination on the stability of Glx-AL at pH 4 (44 °C) (**A**) 7 (54 °C), (**B**) or 9 (58 °C) (**C**) Other specifications are described in Methods section. Glx-AL: open circles; Glx-AL-EDA: solid triangles**.**

Glx-AL was modified with EDA under conditions where the modification of all external carboxylic residues could be expected [50]. This produced a 30% decrease in the activity of the immobilized enzyme (results not shown), while a very small increase in enzyme stability was found in the whole range of studied pH values (Figure 4). Using 58 °C, the residual activity at pH 4 was almost 0 in the first activity measure, while using 44 °C the enzyme remained fully activity after 24 h of inactivation. To calculate with a reasonable guarantee the stabilities of the immobilized enzyme at different pH values, different temperatures were utilized as a function of the enzyme stability. The enzyme has the highest stability at pH 9 and the lowest at pH 4, thus the highest inactivation temperature was used at pH 9 and the lowest at pH 4. The effect of the amination on the enzyme stability was very similar at all pH values, a very small stabilization to that obtained via amination (in fact, not statistically significant). We analysed a wide range of pH to check if changes in the ionic interactions could alter the results and may be very negative in some condition. This very small enzyme stabilization did not compensate the loss of activity. However, it is curious that the difference between the aminated and non-aminated preparations remained almost identical at pH 5, 7 and 9, when it may be expected that the modification on the surface protein interactions may be very different depending on the pH value. Considering Figure 2, there are several areas where ion bridges may be expected, bridges that are now broken and transformed into repulsive interaction. The results suggested that the change of the ionic interactions in the enzyme surface was not very relevant for the enzyme stability.

In any case, this low effect on enzyme activity/stability permitted to use the aminated enzyme in further stabilization strategies (e.g., glutaraldehyde treatment of the Glx-AL-EDA)

### 2.4. Modification with Glutaraldehyde of Alcalase Immobilized in Glyoxyl Agarose Beads (Glx-AL-GLU)

**Figure 5:**
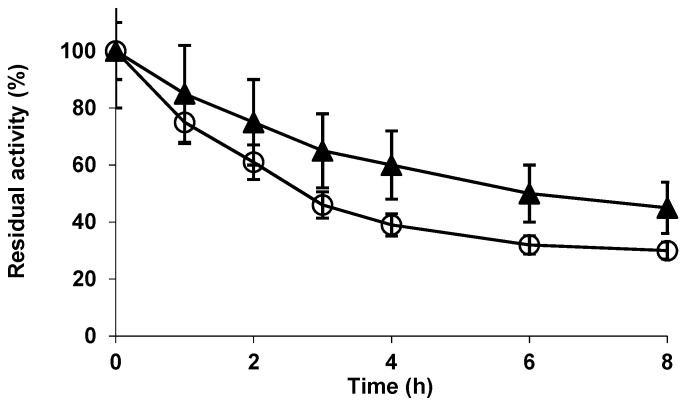
Effect of glutaraldehyde modification on the stability of immobilized Alcalase. The enzyme was modified at pH 7 with 0.1% (*v*/*v*) for 1 h, washed and incubated 3 h at pH 8 before reduction. Inactivation was performed at pH 7 and 54 °C. Other specifications are described in methods. Glx-AL: open circles; Glx-AL-GLU: solid triangles.

The modification of the immobilized Glx-AL with 0.1% glutaraldehyde produced a 30–35% decrease in enzyme activity- Further incubation for 12 h at pH 8 produced a new decrease in enzyme activity, around 50% of the initial activity was preserved after the whole treatment. The immobilized enzyme stability was compared to that of this modified enzyme at pH 7 (Figure 5). A two fold factor increase of the half-life was detected upon glutaraldehyde treatment. That way, it seems that some stabilization could be achieved by glutaraldehyde modification but that this hardly compensates the losses in activity. Looking at Figure 2, only Lys 12 and 15 seems to be near enough to permit a chemical crosslinking and these groups are very likely involved in the enzyme immobilization. Thus, a very intense multi-crosslinking seemed quite unlikely (just Lys 170 and 136 seemed to be near enough).

### 2.5. Modification of Glx-AL-EDA with Glutaraldehyde (Glx-AL-EDA-GLU)

In order to maximize the possibilities of introducing intramolecular crosslinkings via glutaraldehyde chemistry, the aminated enzyme was utilized. Figure 2 shows some areas of the protein quite rich in Asp or Glu (that means that including the terminal carboxyl group there are 11 new amino groups in the protein surface). Even though the exact distances between groups exposed to the medium and after the distortions caused by immobilization and chemical modifications may be different to those of the crystal, some groups are very near and it is possible to speculate that they can participate in some intermolecular crosslinking. For example, Asp 60 and Glu 55, Glu 195, Glu 197 and Asp 172, Asp 172 and Asp 140 are vicinal. Moreover, discarding Lys 12 and 15 (very likely involved in the immobilization), Lys 137 is very near to terminal carboxy group, Lys 27 and Asp 120 are quite close each other; Asp 140 and Lys 136 are also quite near and Lys 170 is surrounded by 3 carboxylic groups. All these can now easily give some intermolecular crosslinkings. This strategy was firstly successfully used to stabilize penicillin G acylase [49], however there are not more reports, it was assayed to ficin but the glutaraldehyde modification of the aminated enzyme drove to the full enzyme inactivation [57].

**Figure 6:**
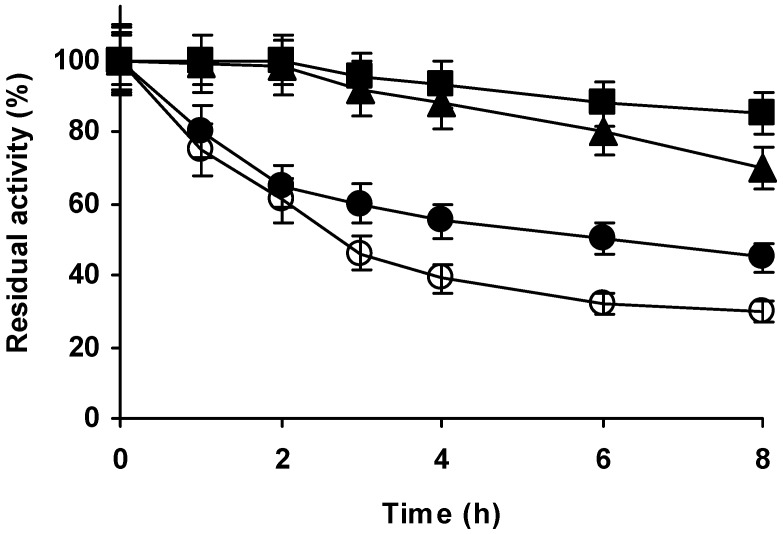
Effect of different glutaraldehyde modifications of Glx-AL-EDA on the inactivation courses at pH 7 and 54 °C. Other specifications are described in methods section. Open circles: aminated enzyme, Solid circles: aminated enzyme modified at pH 7 for 1 h with glutaraldehyde and then reduced; Solid Triangles: aminated enzyme modified with glutaraldehyde at pH 7 for 1 h and then incubated 3 h at pH 8 before reduction, Solid Squares: aminated enzyme modified at pH 7 with glutaraldehyde for 1 h and then incubated at pH 9 for 3 h before reduction.

Thus, the Glx-AL-EDA was treated with 0.1% glutaraldehyde for 1 h. In opposition with the results obtained using ficin, the glutaraldehyde modification of the aminated Alcalase produced just a 20% decrease in enzyme activity, lower than using the non aminated enzyme. This modification permitted to almost fully modify all amino groups in the surface of the aminated enzyme (as confirmed by TNBS titration of reduced biocatalyst). Samples of the immobilized enzymes were incubated for 3 h at pH 8 and pH 9 (this produced a new decrease in enzyme activity by 10% at pH 8 and 15% at pH 9) before reduction to stop the crosslinking reaction. Figure 6 shows that the just modified enzyme was already significantly more stable than the non-modified enzyme at pH 7 but if the enzyme glutaraldehyde modified preparation was incubated for 3 h at pH 9 before reduction, a higher value of stabilization was achieved. As the enzyme just modified with glutaraldehyde was less stable than the enzyme incubated at pH 8 for 3 h and this one was less stable than the enzyme incubated at pH 9, we can assume that a significant percentage of the detected stabilization was achieved by crosslinking involving different reactive amino-glutaraldehyde groups. It should be considered that as the comparison is performed between two immobilized enzyme, autolysis may be discarded [20]. The non-modified enzyme kept 30% of the activity after 8 h, while the immobilized enzyme modified with glutaraldehyde and incubated at pH 9 retained 85% of the initial activity.

**Figure 7:**
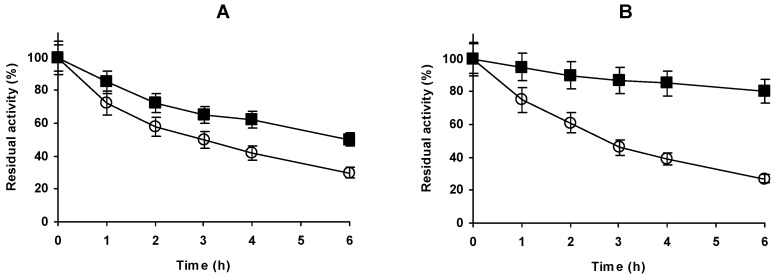
Effect of glutaraldehyde treatment on the stability of immobilized and aminated under different conditions. Glx-AL-EDA was modified at pH 7 with glutaraldehyde and then incubated at pH 9 for 3 h before reduction. Inactivation courses were studied at pH 4 and 44 °C (**A**) and at pH 9 and 58 °C (**B**) Open circles: Glx-AL-EDA, Solid Squares: Glx-AL-EDA-GLU.

Considering in a global way activity and stability, we decided to characterize the modified enzyme incubated at pH 9. Figure 7 shows that the stabilization found at pH 7 could be also detected at pH 9, with very similar values. However, at pH 4 the stabilization was significantly lower (with 30% of activity retention after 6 h for the no modified enzyme and 50% for the modified enzyme).

### 2.6. Hydrolysis of Casein Using Glx-AL-EDA-GLU

**Figure 8:**
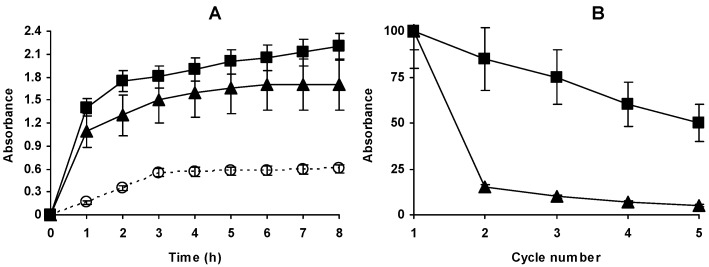
(**A**) Hydrolysis of casein by different preparations of Alcalase at 69 °C and pH 9. Reactions were performed using 0.005 mg of enzyme/mL. Other specifications are described in Methods section. Open circles, dashed line: Free enzyme; Solid triangles: Immobilized Alcalase; Solid Squares: Immobilized, aminated and glutaraldehyde modified enzyme; (**B**) Operational stabilities of different Alcalase preparations at 69 °C and pH 9. Other reaction conditions as in Figure 8A. Solid triangles: Immobilized Alcalase; Triangles, solid line: Immobilized, aminated and glutaraldehyde modified enzyme.

Casein hydrolysis using Glx-AL-EDA-GLU at 50 °C was 30% slower than using Glx-AL (results not shown). However, Figure 8 shows that at 69 °C, Glx-AL-EDA-GLU was 10% more active than Glx-AL and much more active than the free Alcalase. In fact, the free enzyme stopped the reaction far from the hydrolysis level reached by the immobilized preparations, while Glx-AL-EDA-GLU offered a more lineal course. This suggested that the Glx-AL preparation was distorted at this high temperature and for this reason the reaction was stopped before reaching the maximum value. To see if there was a relation between this lower activity and enzyme operational stability, both immobilized enzymes were used for 5 cycles. Figure 7B shows how it is clear that Glx-AL is almost fully inactive just in the second reuse while the Glx-AL-EDA-GLU still kept a high percentage of activity after 5 reuses. Enzyme leakage may be discarded as the secondary amino groups formed after immobilized-enzyme reduction are fully stable, standing even acid lysis of the protein at 110 °C [68].

### 2.7. Reuse of Optimal Preparations at 45 °C and pH 9

**Figure 9:**
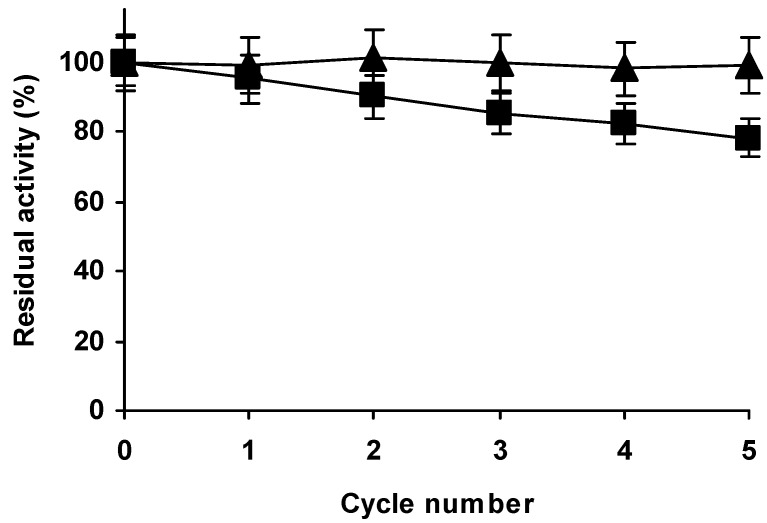
Operational stability of immobilized, aminated and glutaraldehyde treated Alcalase in hydrolysis of casein at pH 9. The experiments were performed at 45 (triangles) or 50 °C (squares). Other experimental conditions are described in methods.

The immobilized enzyme could be used at 69 °C form several cycles (Figure 8B) but the activity was progressively reduced each cycle, making these conditions no recommendable for the enzyme reuse in the long term. Thus, we tried the enzyme reuse under milder conditions. Figure 9 shows the reuses of the glutaraldehyde treated enzyme preparation in hydrolysis of casein at pH 9 and 45 °C or 50 °C. The results show that while at 45 °C the activity remained fully unaltered after 5 reuses (6 cycles of use), at 50 °C more than 20% of initial activity was lost in 6 cycles, therefore the use of the new Alcalase biocatalyst at 45 °C seem convenient from a point of view of enzyme operational activity, although at these temperature the losses of activity caused by the treatment produced a 30% decrease in enzyme activity compared to the unmodified enzyme. The immobilized-only enzyme lost some activity even at 45 °C. Final decision on conditions of use should be taken considering activity and stability, as an accurate way of measuring enzyme stability may be in mass of produced product instead of time of operation [69].

## 3. Materials and Methods 

### 3.1. Materials

Glyoxyl cross-linked 4% agarose beads was from was Agarose Bead Technologies (Torrejón de Ardoz, Spain) and prepared as previously described [64]. Boc-Ala-ONp (NPA) was from Bachem AG (Budendorf, Switzerland). Sodium periodate, glycidol, sodium borohydride, glutaraldehyde (25% (*v*/*v*)), ethyl-3-(3-dimethylaminopropyl)-carbodiimide (EDC), ethylenediamine (EDA), were purchased from Sigma Chemical Co. (St. Louis, MO, USA). Alcalase 2.4 L type FG (liquid form) was a gift from Novo Nordisk A/S (Bagsvaerd, Denmark). All other reagents were of analytical grade.

### 3.2. Alcalase Activity Determination Using Synthetic Substrate

Protease activity of free and immobilized Alcalase was determined spectrophotometrically by the increase in absorbance at 405 nm, produced by the hydrolysis of the synthetic substrate NPA. The reaction was performed in a thermostatized spectrum with continuous stirring using 2 mL of 50 mM sodium phosphate at pH 7, containing 20% ethanol and 20 μL of 100 mM NPA dissolved in acetonitrile at 25 °C (molar extinction coefficient 7843 L mol^−1^ cm^−1^). The immobilized enzyme or an equivalent amount of free enzyme was diluted 1/10 and 50–200 μL were used to determinate the enzyme activity. One NPA unit was defined as the amount of enzyme that hydrolyses 1.0 μmol of NPA per min under the conditions described. The specific activity of the soluble Alcalase was 45 NPA units mg^−1^ of protein in the crude enzyme preparation.

### 3.3. Alcalase Activity Determination Using Casein

The activity versus casein was used, utilizing Kunitz method with slight modifications [70]. A solution of 10 mg/mL of casein was prepared in 100 mM sodium bicarbonate at pH 9.0 at different temperatures from 45 to 69 °C. Then, the desired amount of Alcalase (free or immobilized) was added to 1 mL of substrate solution (ranging from 0.0005 mg to 0.0025 mg) and the reaction mixture was incubated at the indicated temperature, samples being taken periodically. The reaction was stopped by the addition of 1 mL of 10% trichloroacetic acid, incubated for 10 min at room temperature, which precipitated protein molecules but not small peptides and centrifuged at 10,000 rpm. The absorbance of soluble peptides was measured at 280 nm. As reference, the substrate was added after the enzyme was first inactivated by incubation in TCA. One activity unit is defined as the amount of enzyme that increases the absorbance by 0.001 min^−1^ under the given assay conditions.

### 3.4. Alcalase Immobilization on 4 BCL Glyoxyl-Agarose Beads

5 mg of Alcalase were diluted in 100 mL of sodium bicarbonate at pH 10 and 25 °C. Then, 10 g of glyoxyl-agarose beads were added and the reaction was left to proceed for 4 h (this low loading prevent diffusion limitations or enzyme-enzyme interactions) [71]. A reference solution with Alcalase under identical conditions but in the absence of glyoxyl-agarose was used as reference. Periodically, samples of suspension, supernatant and reference were taken and their activities were determined using the NPA assay.

### 3.5. Immobilized Alcalase Amination

Agarose 4BCL beads have been described to immobilized 40 mg of protein/g of support [72]. In this paper, to prevent diffusional limitations and enzyme-enzyme interactions that could alter the results [71], we have used a much lower enzyme loading, 0.5 mg of enzyme/g of support. 10 g of Glx-AL were suspended in 2 M EDA at pH 4.75, then solid EDC was added to a concentration of 10 mM and the suspension was stirred for 90 min. These conditions permitted the full modification of all exposed carboxyl groups of the protein [73,74]. Afterwards, the modified enzyme was washed with an excess of distilled water and stored at 4 °C.

### 3.6. Alcalase Modification with Glutaraldehyde

10 g of Glx-AL or Glx-AL-EDA were suspended in 100 mL of 100 mM sodium phosphate at pH 7 containing 0.1% (*v*/*v*) glutaraldehyde. After 1 h, the preparation was washed to eliminate the free glutaraldehyde and the modified enzyme was incubated at different pH (8 or 9) for 3 h. In all cases, the pH was checked after enzyme addition. At that time, the modified enzyme was incubated for 30 min in 1 mg/mL sodium borohydride to stop the reaction [49].

### 3.7. Alcalase Inactivation

The different Alcalase biocatalysts were suspended at different pH (50 mM sodium acetate at pH 4, sodium phosphate at pH 7 or sodium bicarbonate at pH 9) at temperatures that permitted an inactivation rate adequate to have an accurate determination of the biocatalyst stability, considering the large difference in stability at alkaline and acid pH value. Periodically samples were taken and the activity was determined using the NPA assay.

### 3.8. Immobilized Alcalase Reuses in Hydrolysis of Casein.

After a reaction cycle at pH 9 and 45 °C (0.01 mg of immobilized Alcalase, 10 mg/mL of casein), the enzyme was washed with water and dried in a silica funnel, weighted and re-suspended in a new reaction cycle. 

## 4. Conclusions

Alcalase immobilized on glyoxyl agarose offers some activity versus casein and a significant stabilization, lower using agarose 4BCL than the previously described values using agarose 6BCL, as may be expected from the different geometry of the support [64]. The chemical amination or the modification with glutaraldehyde did not significantly improve enzyme properties; although some stabilization may be found using the individual modifications, it is not so significant as to compensate the decrease in activity. 

This paper shows how the sequential modification of the enzyme with ethylenediamine and treatment with glutaraldehyde significantly improves the enzyme stability after enzyme incubation at pH 9 for 3 h, very likely by permitting an intense intramolecular crosslinking. The native enzyme is relatively poor in Lys residues and all of them are quite far from the other ones as to expect an intense intermolecular crosslinking. The amination greatly increased the number of residues that can participate in the crosslinking, by transforming the external carboxylic groups (some of them previously participating in ion bridges with Lys residues) in primary amine groups. The requirement of an incubation time after glutaraldehyde modification shows that it is not the glutaraldehyde one point modification the responsible of the enzyme improvement on stability but the effect of this incubation suggests that a multi-crosslinking may be obtained, with positive effects on enzyme stability. The modified enzyme can be used even at 69 °C, conditions where the free enzyme was unable even to perform a first cycle. The immobilized and modified enzyme presented very good properties to be used in a diverse range of temperatures. The use of genetic amination may also permit to facilitate the introduction of intermolecular crosslinking, as an alternative to perform in each biocatalysts preparation batch a chemical modification [50].
